# Unique characteristics of end-of-life hospitalizations in Parkinson disease

**DOI:** 10.3389/fnagi.2023.1254969

**Published:** 2023-10-12

**Authors:** Whitley W. Aamodt, Nabila Dahodwala, Warren B. Bilker, John T. Farrar, Allison W. Willis

**Affiliations:** ^1^Department of Neurology, University of Pennsylvania, Philadelphia, PA, United States; ^2^Translational Center of Excellence for Neuroepidemiology and Neurology Outcomes Research, University of Pennsylvania, Philadelphia, PA, United States; ^3^Department of Biostatistics, Epidemiology, and Informatics, University of Pennsylvania, Philadelphia, PA, United States

**Keywords:** Parkinson disease, hospitalization, intensive care unit, length of stay, cost, mortality

## Abstract

**Introduction:**

Persons with Parkinson disease (PD) are hospitalized at higher rates, have longer lengths of stay, and are more likely to die in the hospital than age-matched peers. Although prior studies have compared inpatient outcomes between persons with and without PD, little is known about inpatient outcomes across the PD trajectory, or whether hospitalizations occurring in the last 6 months of life differ from earlier hospitalizations.

**Methods:**

This cross-sectional study compared Medicare Part A and B beneficiaries aged 65 and older with a qualifying PD diagnosis who were hospitalized in 2017: decedents who died between 7/1/2017 and 12/31/2017 from all causes and were hospitalized at least once in their last 6 months of life, and non-decedents who were hospitalized between 1/1/2017 and 6/30/2017 and lived 6 or more months after discharge. End-of-life (EoL) hospitalizations were defined as those occurring in the last 6 months of life. Descriptive analyses compared patient-level variables (e.g., demographics, comorbidities, treatment intensity) and encounter-level variables (e.g., length of stay, total charges) between groups. Multivariable logistic regression models also compared rates of intensive care unit (ICU) admission and 30-day readmission between hospitalized decedents and hospitalized non-decedents, adjusting for age, sex, race/ethnicity, rural residence, and Charlson Comorbidity Index Score.

**Results:**

Of 26,492 Medicare decedents with PD, 16,187 (61.1%) were hospitalized in their last 6 months of life. Of 347,512 non-decedents with PD, 62,851 (18.1%) were hospitalized in a 6-month period. Hospitalized decedents were slightly older than hospitalized non-decedents (82.3 [SD 7.40] vs. 79.5 [SD 7.54] years) and had significantly more comorbidities. Compared to non-EoL hospitalizations, EoL hospitalizations were slightly longer (5 [IQR 3–9] vs. 4 [IQR 3–7] days) and more expensive based on total charges per admission ($36,323 [IQR 20,091-69,048] vs. $32,309 [IQR 18,789–57,756]). In covariate-adjusted regression models using hospitalized non-decedents as the reference group, hospitalized decedents were more likely to experience an ICU admission (AOR 2.36; CI 2.28–2.45) and 30-day readmission (AOR 2.43; CI 2.34–2.54).

**Discussion:**

Hospitalizations occurring in the last 6 months of life among persons with PD in the United States are longer, more costly, and more resource intensive than earlier hospitalizations and may stem from medical comorbidities. Once hospitalized, ICU admission and 30-day readmission may aid in prognostication and serve as markers of transition to the EoL period.

## Introduction

Parkinson disease (PD) is the second most common neurodegenerative disorder worldwide and the 14th leading cause of death in the United States (Marras et al., 2018; Kochanek et al., 2019). There are no disease-modifying therapies for PD, and progressive symptoms contribute to significant morbidity and mortality. Not surprisingly, age and advancing symptoms increase the risk of hospitalization (Woodford and Walker, 2005; Hobson et al., 2012; Hassan et al., 2013), and persons with PD are hospitalized at higher rates, have longer lengths of stay, and are more likely to die in the hospital than age-matched peers (Aminoff et al., 2011; Gerlach et al., 2012; Low et al., 2015; Shahgholi et al., 2017). Despite these data, little is known about PD hospitalizations in the end-of-life (EoL) period, commonly defined as the last 6 months of life (Lamont, 2005; Hui et al., 2014).

A recent study exploring inpatient treatment intensity among hospitalized patients with PD found that the final 6 months of life are a critical period in PD care (Aamodt et al., 2023a). Almost two-thirds of all United States Medicare beneficiaries with PD are hospitalized at least once in their last 6 months of life with high rates of aggressive care (Aamodt et al., 2023a). For example, of 33,107 beneficiaries with PD who were hospitalized in the EoL period in 2017, 16,266 (49%) were transferred to the intensive care unit (ICU) and 7,970 (25%) died in the hospital. Of those surviving hospitalization, 9,892 (30%) were discharged to inpatient or home hospice and 10,046 (30%) were readmitted within 30 days. These inpatient outcomes are considered markers of inappropriate EoL care quality in persons with Alzheimer’s disease (AD) and may suggest inappropriate EoL care practices in PD (De Schreye et al., 2017). However, it is currently unknown whether increased rates of hospitalization or inpatient treatment intensity among persons with PD are unique to the EoL period or occur throughout the disease course.

Building upon our existing data, the primary objectives of the current study were to (1) describe and compare the demographic and clinical characteristics of United States Medicare beneficiaries with PD who were hospitalized at least once in their last 6 months of life (e.g., hospitalized decedents) or hospitalized at least once before the EoL period (e.g., hospitalized non-decedents), (2) compare inpatient resource utilization and total inpatient charges between hospitalized decedents and non-decedents, and (3) determine whether rates of ICU admission and 30-day readmission differ between hospitalized decedents and non-decedents after adjusting for key demographic and clinical variables.

## Methods

### Protocol approval

This study was approved by the University of Pennsylvania Human Research Protections Office and the Centers for Medicare & Medicaid Services (CMS) via a Data Use Agreement and waiver of consent. Data analysis was conducted from January 2023 to April 2023.

### Data source

The Medicare program insures more than 98% of United States adults aged 65 and older and provides inpatient, outpatient, and prescription drug coverage (Research Data Assistance Center, 2015). The current study used data from the 2017 Master Beneficiary Summary File (MBSF), 2017 Medicare Provider Analysis and Review (MedPAR) file, and 2015–2017 Carrier files. CMS files contain individual-level data that can be linked across datasets using a unique beneficiary identification code. The MBSF contains Medicare enrollment and eligibility information, validated birth and death dates, demographic data, postal codes, and indicator variables for 27 common chronic medical conditions obtained using validated algorithms (Chronic Conditions Data Warehouse, 2023). The MedPAR file contains information on 100% of Medicare beneficiaries admitted to acute care hospitals and skilled nursing facilities covered by Medicare. These files contain admission and discharge dates, admission and principal diagnoses, procedure codes, total charges, and reimbursement amounts. The Carrier files contain fee-for-service claims submitted by professional providers, including physicians, physician assistants, nurse practitioners, and clinical social workers. The MedPAR and Carrier files contain International Classification of Diseases, Ninth Revision (ICD-9) codes for services provided before October 1, 2015, and International Classification of Diseases, Tenth Revision (ICD-10) codes for services provided on or after October 1, 2015.

### Inclusion and exclusion criteria

We conducted a population-based cross-sectional study comparing two cohorts of Medicare Part A (inpatient) and Part B (outpatient) beneficiaries aged 65 and older with at least two validated inpatient and/or outpatient encounter diagnoses for PD (ICD-9 code 332 [paralysis agitans]; ICD-10 code G20 [Parkinson disease]) between January 1, 2015 and December 31, 2017. Hospitalized decedents with PD were defined as individuals with a validated death date between July 1, 2017 and December 31, 2017 from all causes who were hospitalized at least once in their last 6 months of life. Hospitalized non-decedents with PD were individuals who were hospitalized between January 1, 2017 and June 30, 2017 and lived 6 or more months after discharge. These dates were chosen to restrict analyses to a single year of data and allow for cost comparisons. Persons enrolled in both Medicare and Medicaid services, known as dual-eligible beneficiaries, were also included, as Medicare remains the primary source of financing for acute care services (Liu et al., 2006). We excluded Medicare Advantage (Part C) beneficiaries with private insurance benefits whose inpatient care patterns would not be reflected in CMS data, along with beneficiaries who had diagnostic codes for atypical or secondary parkinsonism (ICD-9 code 332.1 [drug-induced parkinsonism], 333.0 [atypical parkinsonism], 094.82 [syphilitic parkinsonism]; ICD-10 code G21 [secondary parkinsonism], G21.0 [neuroleptic malignant syndrome], G21.1 [other drug-induced secondary parkinsonism], G21.2 [secondary parkinsonism due to other external agents], G21.3 [post-encephalitic parkinsonism], G21.4 [vascular parkinsonism], G21.8 [other secondary parkinsonism], G21.9 [secondary parkinsonism, unspecified]).

### Study outcomes

Primary study outcomes in descriptive analyses included patient-level variables (e.g., demographics, comorbidities, treatment intensity) and encounter-level variables (e.g., hospital length of stay, total charges), while secondary outcomes included discharge disposition. Primary study outcomes in logistic regression models included ICU admission and 30-day readmission, chosen because they are common among persons with PD in the EoL period (Aamodt et al., 2023a) and considered markers of inappropriate EoL care quality in persons with AD (De Schreye et al., 2017), another neurodegenerative disorder.

### Covariates

We extracted demographic data and comorbidities to create covariates and stratification variables based on age, sex, race/ethnicity, rural residence, and Charlson Comorbidity Index (CCI) score (Charlson et al., 1987; Quan et al., 2005). Age was calculated based on birth date. Sex is dichotomized in CMS files as “male” or “female.” Race and ethnicity are mutually exclusive categories and were categorized alphabetically as “Asian,” “Black,” “Hispanic,” “Native North American,” “Unknown/Other,” and “White.” The recorded county of the beneficiary mailing address was used to determine the location of residence in a rural or urban area based on the 2013 United States Department of Agriculture Rural–Urban Continuum Codes, a classification scheme that subdivides counties into 6 nonmetro (rural) and 3 metro (urban/suburban) areas (USDA ERS - Documentation, 2022). Medical comorbidity burden was calculated using the CCI.

### Statistical analysis

First, descriptive statistics were used to characterize and compare hospitalized decedents and hospitalized non-decedents with PD. Second, logistic regression models were built to determine whether demographic variables, CCI, or the timing of a hospitalization (e.g., EoL vs. non-EoL) were associated with primary outcomes among PD patients admitted to acute care hospitals. Univariable models examined the relationship between primary outcomes and individual patient factors (e.g., age, sex, race/ethnicity, rural residence, and medical comorbidities). Multivariable models examined the odds of each primary outcome after adjusting for a combination of patient factors. All statistical tests were two-sided with an alpha level of 0.05. Analyses were conducted using Stata (v17.0; StataCorp, College Station, TX).

## Results

### Demographic and clinical characteristics

**Figure 1:**
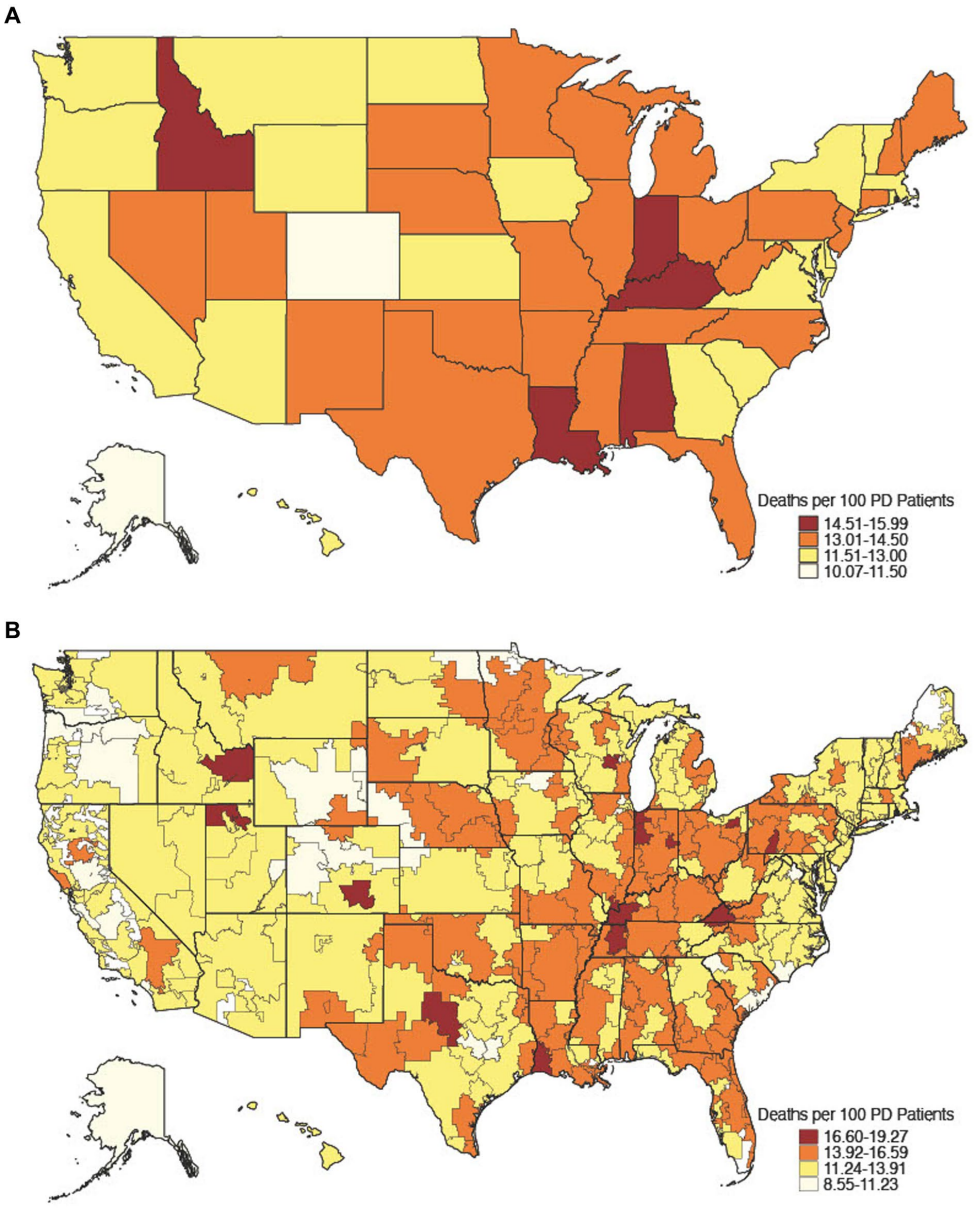
Age-adjusted all-cause mortality rate, 2017. All-cause mortality rate among 2017 Medicare decedents aged 65 and older by state [**(A)** *n* = 53,279] and hospital referral region [**(B)** *n* = 53,162], a geographical unit derived from the Dartmouth Atlas of Health Care that defines 306 unique Medicare healthcare market regions composed of 5-digit zip code areas grouped by referral patterns for tertiary care (The Center for the Evaluative Clinical Sciences, Dartmouth Medical School, 1996).

There were 400,791 Medicare beneficiaries aged 65 and older with PD in 2017, of which 53,279 died, yielding an age-adjusted all-cause mortality rate of 13.3% with geographic variability (Figure 1). Of decedents with PD, 26,492 (49.7%) died between July 1, 2017, and December 31, 2017, of which 16,187 (61.1%) were hospitalized at least once in their last 6 months of life and formed our hospitalized decedent cohort. Of 347,512 non-decedents with PD, 62,851 (18.1%) were hospitalized at least once between January 1, 2017, and June 30, 2017 and lived at least 6 months after discharge and formed our hospitalized non-decedent cohort (Table 1). The mean age of hospitalized decedents and hospitalized non-decedents was 82.3 (SD 7.40) years and 79.5 (SD 7.54) years, respectively. In both cohorts, beneficiaries were predominantly male, White, and most likely to live in suburban/urban areas. Hospitalized decedents were also more likely to be dual-eligible for Medicare and Medicaid services due to disability or poverty (5,272; 32.6%) than hospitalized non-decedents (18,156; 28.9%). More than one-third of hospitalized decedents with PD had 6 or more chronic conditions (6,048; 37.4%) compared to a quarter of hospitalized non-decedents (17,541; 27.9%). Notably, hospitalized decedents with PD were more likely to be diagnosed with dementia (13,644; 84.3%) than hospitalized non-decedents (43,130; 68.6%). Demographic and clinical data are summarized in Table 1.

**Table 1:** Demographic and clinical characteristics.

	Hospitalized decedents (*n* = 16,187)	Hospitalized non-decedents^b^ (*n* = 62,851)
**Age, years; mean (SD)**	82.30 (7.40)	79.46 (7.54)
Male	81.72 (7.19)	78.93 (7.34)
Female	83.09 (7.63)	80.05 (7.71)
**Age, years; *n* (%)**		
65–69	876 (5.41)	6,832 (10.87)
70–74	1,809 (11.18)	11,060 (17.60)
75–79	2,918 (18.03)	13,777 (21.92)
80–84	3,903 (24.11)	13,842 (22.02)
85–89	3,944 (24.37)	11,146 (17.73)
>90	2,737 (16.91)	6,194 (9.86)
**Sex, *n* (%)**		
Male	9,674 (59.76)	33,396 (53.14)
Female	6,513 (40.24)	29,455 (46.86)
**Race/Ethnicity, *n* (%)**		
White	14,292 (88.29)	55,233 (87.88)
Black	1,014 (6.26)	3,800 (6.05)
Hispanic	282 (1.74)	1,147 (1.82)
Asian	257 (1.59)	1,023 (1.63)
North America Native	71 (0.44)	300 (0.48)
Unknown/Other	271 (1.67)	1,348 (2.14)
**Region, *n* (%)**		
Northeast	3,115 (19.24)	12,405 (19.74)
Midwest	3,706 (22.89)	14,465 (23.01)
South	6,797 (41.99)	25,542 (40.64)
West	2,535 (15.66)	10,334 (16.44)
Other	34 (0.21)	105 (0.17)
**Rurality, *n* (%)** ^**a**^		
Urban/suburban	12,910 (79.76)	50,190 (79.86)
Rural	3,262 (20.15)	12,489 (19.87)
Unknown	15 (0.09)	172 (0.27)
**Dual eligibility, *n* (%)**		
No	10,915 (67.43)	44,695 (71.11)
Yes	5,272 (32.57)	18,156 (28.89)
**Part D coverage, *n* (%)**		
No	3,842 (23.74)	14,297 (22.75)
Yes	12,345 (76.26)	48,554 (77.25)
**Chronic condition count, *n* (%)**		
0–1	678 (4.19)	6,349 (10.10)
2–3	3,431 (21.20)	16,595 (26.40)
4–5	6,030 (37.25)	22,366 (35.59)
≥6	6,048 (37.36)	17,541 (27.91)
**Chronic conditions, *n* (%)**		
Alzheimer disease and related disorders or senile dementia	13,644 (84.29)	43,130 (68.62)
Atrial fibrillation	6,289 (38.85)	20,152 (32.06)
Cancer	5,498 (33.97)	16,933 (26.94)
Prostate	2,020 (12.48)	6,175 (9.82)
Breast	903 (5.58)	3,699 (5.89)
Colorectal	949 (5.86)	2,757 (4.39)
Leukemia/lymphoma	732 (4.52)	2,152 (3.42)
Lung	701 (4.33)	1,451 (2.31)
Endometrial	193 (1.19)	759 (1.21)
Chronic obstructive pulmonary disease	8,488 (52.44)	29,604 (47.10)
Ischemic heart disease	12,989 (80.24)	47,378 (75.38)
Congestive heart failure	10,715 (66.20)	35,385 (56.30)
Diabetes mellitus	9,101 (56.22)	34,545 (54.96)
End-stage renal disease	372 (2.30)	1,020 (1.62)
Liver disease/cirrhosis	2,916 (18.01)	10,316 (16.41)
Stroke/TIA	7,595 (46.92)	26,196 (41.68)
**Mood disorders, *n* (%)**		
Depression	10,464 (64.64)	40,289 (64.10)
Anxiety	8,978 (55.46)	35,439 (56.39)
Schizophrenia/psychosis	4,612 (28.49)	15,242 (24.25)

Medicare decedents with Parkinson disease died between July 1, 2017 and December 31, 2017 and were hospitalized at least once in their last 6 months of life, while non-decedents with Parkinson disease were hospitalized at least once between January 1, 2017 and June 30, 2017 and lived 6 or more months after discharge. ^a^Rural/urban residence was determined using 2013 US Department of Agriculture Rural–Urban Continuum Codes (RUCC), a classification scheme that subdivides counties into 6 nonmetro (rural) and 3 metro (urban) groupings based on 5-digit Federal Information Processing Standard (FIPS) code. ^b^Hospitalized decedents and hospitalized non-decedents with Parkinson disease were compared using independent sample *t*-tests for normally distributed continuous variables and Chi-square analyses for categorical variables. Due to the large sample size, all comparisons were significantly different (*p* < 0.05) with the exception of the following: race/ethnicity; residence in the Northeast and Midwest; rurality; Part D coverage; 4–5 chronic conditions; and prior diagnosis of breast cancer, endometrial cancer, and depression. SD, standard deviation; TIA, transient ischemic attack.

### Inpatient resource utilization and total charges

Of the 26,492 Medicare decedents with PD who died between July 1, 2017, and December 31, 2017, 16,187 (61.1%) were hospitalized at least once in their last 6 months of life, resulting in 31,415 admissions. Of the 347,512 non-decedents with PD, 62,851 (18.1%) were hospitalized at least once between January 1, 2017, and June 30, 2017, and lived at least 6 months after discharge, resulting in 93,478 admissions (Table 2). The five most common inpatient principal diagnoses for hospitalized decedents were sepsis, aspiration pneumonitis, urinary tract infection (UTI), acute kidney failure, and PD. In comparison, the five most common inpatient principal diagnoses for hospitalized non-decedents were sepsis, PD, UTI, acute kidney failure, and pneumonia (Supplementary Table S1).

**Table 2:** Inpatient treatment intensity, discharge disposition, and total charges.

	Hospitalized decedents	Hospitalized non-decedents
**(A). Total patients admitted, *n* (%)**	16,187 (61.1)	62,851 (18.1)
ICU admission	8,010 (49.5)	18,047 (28.7)
Invasive mechanical ventilation	1,575 (9.7)	813 (1.3)
Percutaneous feeding tube	475 (2.9)	497 (0.8)
Blood transfusion	610 (3.8)	943 (1.5)
30-Day readmission	4,881 (30.2)	9,204 (14.6)
Hospice discharge	4,801 (29.7)	599 (1.0)
Discharged to home hospice	2,074 (12.8)	427 (0.7)
Discharged to inpatient hospice	2,727 (16.8)	172 (0.3)
In-hospital death	3,892 (24.0)	–
**(B). Total admissions, # (%)**	31,415	93,478
ICU admission	11,132 (35.4)	21,592 (23.1)
Invasive mechanical ventilation	1,803 (5.7)	918 (1.0)
Percutaneous feeding tube	476 (1.5)	510 (0.5)
Blood transfusion	696 (2.2)	1,014 (1.1)
Length of stay in days, median (IQR)	5 (3–9)	4 (3–7)
30-Day readmission	7,491 (23.8)	12,227 (13.1)
Discharged to home/self-care	3,201 (10.2)	26,947 (28.8)
Discharged to inpatient rehabilitation	1,041 (3.3)	6,242 (6.7)
Discharged to skilled nursing	11,358 (36.2)	32,147 (34.4)
Discharged to LTAC	685 (2.2)	869 (0.9)
Discharged to other care	6,385 (20.3)	26,652 (28.5)
Discharged to home hospice	2,112 (6.7)	445 (0.5)
Discharged to inpatient hospice	2,741 (8.7)	176 (0.2)
In-hospital death	3,892 (12.4)	--
Admissions/100 decedents or beneficiaries	118.6	26.9
**(C). Total charges, USD**	1,935,551,428	4,606,930,619
Charges/admission, median (IQR)	36,323 (20,091-69,048)	32,309 (18,789-57,756)

Individual-level (A) and encounter-level (B) data on inpatient treatment intensity and discharge disposition, along with total inpatient charges (C). Of 26,492 Medicare decedents who died between July 1, 2017 and December 31, 2017, 16,187 were hospitalized at least once in their last 6 months of life. Of 347,512 non-decedents in 2017, 62,851 were hospitalized at least once between January 1, 2017 and June 30, 2017 and lived 6 or more months after discharge. ICU, intensive care unit; IQR, interquartile range; LTAC, long-term acute care hospital; USD, United States dollar.

Of the 16,187 PD decedents admitted to the hospital in the last 6 months of life, 8,010 (49.5%) had at least one episode of ICU care, 1,575 (9.7%) were mechanically ventilated, 475 (2.9%) received a percutaneous feeding tube, 610 (3.8%) received a blood transfusion, 4,881 (30.2%) were readmitted to the hospital within 30 days, 4,801 (29.7%) were discharged to hospice care, and 3,892 (24.0%) died in the hospital. Of the 62,851 PD decedents admitted to the hospital before the EoL period, 18,047 (28.7%) had at least one episode of ICU care, 813 (1.3%) were mechanically ventilated, 497 (0.8%) received a percutaneous feeding tube, 943 (1.5%) received a blood transfusion, 9,204 (14.6%) were readmitted to the hospital within 30 days, 599 (1.0%) were discharged to hospice care, and 0 (0.0%) died in the hospital.

Because some decedents were hospitalized multiple times, other outcomes are reported as encounter-level data (Table 2). Compared to non-EoL hospitalizations, EoL hospitalizations resulted in a slightly longer median length of stay (5 days [IQR 3–9] vs. 4 days [IQR 3–7]) and greater median total charges per admission in United States dollars ($36,323 [IQR 20,091–69,048] vs. $32,309 [IQR 18,789–57,756]). Discharge dispositions are summarized in Table 2.

### ICU admission and 30-day readmission

Next, univariable and multivariable logistic regression models were used to estimate the relative odds of each primary outcome occurring in hospitalized decedents and hospitalized non-decedents with PD, adjusting for a combination of age, sex, race/ethnicity, rural/urban residence, and CCI (Table 3). In adjusted models using hospitalized non-decedents as the reference group, those hospitalized in the last 6 months of life had greater odds of ICU admission (AOR 2.36; CI 2.28–2.45) and 30-day readmission (AOR 2.43; CI 2.34–2.54) with notable sex, racial, ethnic, and geographic differences that have been previously reported (Aamodt et al., 2023a). We did not perform regression analyses on demographic, clinical, or encounter-level variables.

**Table 3:** Frequency and relative odds of ICU admission and 30-day readmission.

	Frequency *n* (%)	Unadjusted OR	95% CI	Adjusted OR^a^	95% CI
**(A). ICU admission (*n* = 26,057)**					
Admission type					
Non-decedent (>6 months)	18,047 (69.3)	ref	ref	**ref**	**ref**
Decedent (<6 months)	8,010 (30.7)	**2.43**	**2.35–2.52**	**2.36**	**2.28–2.45**
Age	26,057 (100.0)	1.00	0.99–1.00	**0.99**	**0.98–0.98**
Sex					
Male	14,975 (57.5)	ref	ref	ref	ref
Female	11,082 (42.5)	**0.84**	**0.81–0.86**	**0.91**	**0.88–0.94**
Race/Ethnicity					
White	22,539 (86.5)	ref	ref	ref	ref
Black	1,772 (6.8)	**1.16**	**1.09–1.23**	1.03	0.96–1.10
Hispanic	587 (2.3)	**1.45**	**1.31–1.62**	**1.37**	**1.23–1.53**
Asian	526 (2.0)	**1.45**	**1.30–1.63**	**1.42**	**1.26–1.59**
North America Native	116 (0.5)	0.95	0.76–1.18	1.03	0.82–1.29
Unknown/Other	567 (2.2)	**1.12**	**1.01–1.25**	1.11	0.99–1.23
Rural Residence	4,701 (18.0)	**0.84**	**0.80–0.87**	**0.86**	**0.82–0.89**
Charlson Comorbidity Index	26,057 (100.0)	**1.10**	**1.09–1.10**	**1.09**	**1.08–1.09**
**(B). 30-Day readmission (*n* = 14,085)**					
Admission type					
Non-decedent (>6 months)	9,204 (65.4)	ref	ref	ref	ref
Decedent (<6 months)	4,881 (34.6)	**2.52**	**2.42–2.62**	**2.43**	**2.34–2.54**
Age	14,085 (100.0)	**0.99**	**0.99–0.99**	**0.98**	**0.98–0.99**
Sex					
Male	7,853 (55.8)	ref	ref	ref	ref
Female	6,232 (44.2)	**0.94**	**0.91–0.97**	**1.06**	**1.02–1.10**
Race/Ethnicity					
White	12,171 (86.4)	ref	ref	ref	ref
Black	1,064 (7.6)	**1.34**	**1.25–1.44**	**1.12**	**1.04–1.21**
Hispanic	302 (2.1)	**1.26**	**1.11–1.44**	**1.15**	**1.01–1.21**
Asian	214 (1.5)	0.95	0.82–1.10	0.90	0.77–1.05
North America Native	70 (0.5)	1.10	0.84–1.42	1.15	0.89–1.50
Unknown/Other	264 (1.9)	0.92	0.80–1.05	0.90	0.79–1.04
Rural Residence	2,681 (19.0)	**0.93**	**0.89–0.98**	0.96	0.92–1.01
Charlson Comorbidity Index	14,085 (100.0)	**1.14**	**1.14–1.15**	**1.13**	**1.12–1.14**

Frequency and relative odds of intensive care unit admission (A) and 30-day readmission (B) in hospitalized Medicare beneficiaries with Parkinson disease. ^a^Multivariable regressions were adjusted for a combination of age, sex, race/ethnicity, rural/urban residence, and Charlson Comorbidity Index score. Statistically significant ORs are in bold. ICU, intensive care unit; OR, odds ratio; CI, confidence interval; ref, reference group for logistic regression model.

## Discussion

In this study using comprehensive Medicare data from 2017, we found that persons with PD who were hospitalized in the last 6 months of life were slightly older and had more medical comorbidities than hospitalized persons who survived 6 or more months after discharge. Compared to non-EoL hospitalizations, EoL hospitalizations among persons with PD were also longer and more costly. Notably, PD patients hospitalized in their last 6 months of life had twice the odds of ICU admission and 30-day readmission as those hospitalized at earlier time points after controlling for key demographic and clinical variables. Consistent with prior studies, these data suggest that the last 6 months of life are a critical period in PD care and associated with significant, potentially preventable healthcare utilization (McKenzie et al., 2022; Aamodt et al., 2023a).

When comparing the clinical characteristics of hospitalized decedents and hospitalized non-decedents, decedents in our PD cohort were more likely to have 6 or more medical comorbidities and had increased rates of dementia, which likely contributed to group differences in hospitalization rates (Phelan et al., 2012). Not surprisingly, age and comorbidity burden are associated with an increased risk of all-cause hospitalization and mortality in older adults (Miller et al., 1998) and persons with PD (Braga et al., 2014). Hospitalizations among PD patients can also lead to hospital-related complications that may hasten death (Aminoff et al., 2011; Gerlach et al., 2012; Low et al., 2015), underscoring the need to ensure that all hospitalizations are medically necessary and aligned with care preferences. Thus, a multi-disciplinary approach involving general, neurological, and palliative care providers is required to prevent inappropriate hospitalizations whenever possible.

Next, when comparing inpatient resource utilization between hospitalized decedents and hospitalized non-decedents admitted for all causes, inpatient treatment intensity was significantly greater among decedents, with longer lengths of stay and more costly admissions in the last 6 months of life. Our findings support results from prior studies which also showed that persons with PD have longer lengths of stay than individuals in the general population (Gerlach et al., 2011). Longer hospital stays in PD patients have been associated with delayed or missed administration of dopaminergic drugs (Martinez-Ramirez et al., 2015), administration of dopamine receptor blocking agents (Martinez-Ramirez et al., 2015), elective surgery (Kleiner et al., 2019), and post-operative delirium and other complications (Pepper and Goldstein, 1999; Abboud et al., 2020). However, further research is needed to determine risk factors for prolonged length of stay in the EoL period. With regard to cost, hospitalizations account for the greatest proportion of EoL healthcare spending relative to other spending categories (French et al., 2017). In the United States, Medicare spending in the last year of life accounts for up to 25% of total Medicare costs (Duncan et al., 2019), and increased spending at EoL may reflect inappropriate care practices. Hospitalizations in the last 6 months of life may also be incongruent with care preferences, reiterating the importance of advance care planning for persons with PD to improve quality of life and reduce spending at the national level.

Lastly, we found that hospitalized decedents with PD were twice as likely as hospitalized non-decedents with PD to be admitted to the ICU or experience a 30-day readmission after controlling for age, sex, race/ethnicity, rural/urban residence, and comorbidities. ICU admissions among persons with PD are often unrelated to PD duration or severity (Paul et al., 2019), and ICU mortality data are overall mixed. In one study comparing ICU admissions between persons with and without PD in Brazil, those with PD had longer hospital stays but did not experience an increased mortality risk resulting from their ICU admission (Réa-Neto et al., 2021). However, in a second study involving a random sample of elderly Medicare beneficiaries, ICU length of stay was associated with an increased risk of 1-year mortality among ICU survivors (Moitra et al., 2016). Hospital readmission is also common among persons with PD (Shahgholi et al., 2017). Although we could not determine the exact reasons for readmission in our cohort, other studies have demonstrated that readmissions among PD patients are associated with medical comorbidities, elective surgeries, and caregiver strain (Shahgholi et al., 2017; Fullard et al., 2020). For example, persons with advanced PD become increasingly reliant on caregivers for support, and increasing physical dependence can lead to caregiver burden (Lo Monaco et al., 2021; Aamodt et al., 2023b). Caregiver burden and depression are associated with a higher risk of emergency department visits (Rashid et al., 2023) and re-hospitalization among persons with PD (Shahgholi et al., 2017), presumably when caregivers are overwhelmed and require additional support. In the general population, 30-day readmission is also associated with age, male sex, comorbidities, polypharmacy, and length of stay at the initial hospital visit (Glans et al., 2020). Because ICU admission and 30-day readmission among persons with PD are significantly more common in the last 6 months of life, these hospital outcomes may signify a transition to the EoL period and aid in prognostication. Interestingly, the principal admission diagnosis was similar between hospitalized decedents and hospitalized non-decedents and may not be a reliable indicator of the EoL period.

Numerous studies have advocated for improved EoL care in the United States (Teno et al., 2013; Committee on Approaching Death: Addressing Key End of Life Issues, Institute of Medicine, 2015). Although rates of EoL hospitalization declined among United States Medicare beneficiaries between 2000 and 2015 (Emanuel, 2018; Teno et al., 2018), progress is slow and EoL interventions are needed for those at greatest risk of death. Despite the prognostic uncertainty associated with heterogeneous symptoms and rates of disease progression in persons with PD, our findings have important clinical implications. Because PD patients hospitalized in their last 6 months of life have twice the odds of ICU admission and 30-day readmission, these events could trigger automatic inpatient palliative care consultations to clarify goals of care. While advance care planning can reduce unwanted, invasive, and potentially inappropriate EoL care (Kwak et al., 2014), most discussions occur in the outpatient setting and may miss critical opportunities to intervene after hospital admission. Thus, inpatient admissions can offer time-sensitive opportunities to discuss EoL preferences and prompt earlier referral to hospice care when indicated and desired.

This study has several limitations. First, Medicare is an administrative dataset, and studies using Medicare claims data are limited to the diagnoses and treatments documented in the medical record and subject to misclassification bias. Second, although comorbid dementia was determined using a validated indicator variable for Alzheimer’s disease and related disorders (Chronic Conditions Data Warehouse, 2023), the exact dementia diagnosis was unknown. Similarly, the cause of death among hospitalized decedents in our cohort was unknown, and hospitalizations among persons with PD may stem from other illnesses. However, sepsis, aspiration pneumonitis, UTI, acute kidney failure, and PD comprised the top 31% of inpatient principal diagnoses among hospitalized decedents. By contrast, these conditions only comprised the top 20% of inpatient principal diagnoses among hospitalized non-decedents (Supplementary Table S1), suggesting that hospitalizations in the EoL period are more often associated with PD-related system failure. Because UTIs and pneumonia are leading causes of acute hospitalization in persons with advanced PD (Okunoye et al., 2020), further work is needed to minimize these risk factors. Future studies should also explore inpatient outcomes at EoL using more granular clinical data. In addition, we could not account for a do-not-resuscitate (DNR) order or pre-existing advance directives that may have influenced resource utilization and treatment intensity. While hospitalized patients with PD have higher odds of DNR utilization than other hospitalized patients (Mahajan et al., 2017), hospitalized decedents in our cohort still had high rates of treatment intensity and readmission, suggesting that new approaches to advance care planning may be needed in acute care settings. Lastly, our study presents data from a large, nationally representative sample of persons with PD who were hospitalized before the COVID-19 pandemic. Future studies should also utilize more recent data, when available, to account for post-pandemic changes in healthcare delivery, acute care utilization, and life expectancy (Balser et al., 2021; Pujolar et al., 2022; Schöley et al., 2022; Huang et al., 2023). Despite these limitations, this study is among the first to describe differences in inpatient outcomes across the PD lifespan.

In conclusion, intensive and frequent hospitalizations among persons with PD are a unique characteristic of the EoL period. Although hospitalizations may stem from non-PD-related illnesses, inpatients with PD are twice as likely to experience an ICU admission and hospital readmission in the last 6 months of life than at other time points. Because hospitalizations among persons with PD may portend a poor prognosis and reflect poor EoL care quality, providers must ensure that all hospitalizations are reasonable, necessary, and aligned with care preferences. In addition, because caregiver burden is a risk factor for re-hospitalization among persons with PD, inpatient admissions may provide important opportunities to address caregiver strain. Further work is needed to reduce hospitalizations in the EoL period, which can lead to improved quality of life, more efficient resource allocation, and reduced healthcare spending for all persons with PD.

## Data availability statement

The data analyzed in this study is subject to the following licenses/restrictions: all datasets are available to purchase at resdac.org/. Aggregated de-identified data may be shared on request. Requests to access these datasets should be directed to https://resdac.org/.

## Ethics statement

The studies involving humans were approved by the University of Pennsylvania Human Research Protections Office and the Centers for Medicare and Medicaid Services via a Data Use Agreement and waiver of consent. The studies were conducted in accordance with the local legislation and institutional requirements. Written informed consent for participation was not required from the participants or the participants’ legal guardians/next of kin in accordance with the national legislation and institutional requirements.

## Author contributions

WA: Conceptualization, Data curation, Formal analysis, Methodology, Writing – original draft, Writing – review & editing. ND: Conceptualization, Formal analysis, Methodology, Writing – review & editing. WB: Data curation, Methodology, Writing – review & editing. JF: Conceptualization, Methodology, Supervision, Writing – review & editing. AW: Conceptualization, Data curation, Formal analysis, Methodology, Supervision, Writing – review & editing.
