# Supplementary material for: Unique characteristics of end-of-life hospitalizations in Parkinson disease
Source: Front Aging Neurosci. 2023 Oct 12;15:1254969. doi: 10.3389/fnagi.2023.1254969 (PMC10600520; doi:10.3389/fnagi.2023.1254969)
Supplement: Supplementary file 1 [file Table_1.DOCX]

**Supplemental Table**

Top 10 ICD-10 diagnostic codes for inpatient admission diagnoses (A) and inpatient principal diagnoses (B) among hospitalized decedents and non-decedents with PD.

A. Admission Diagnoses

| Hospitalized Decedents (31,415 admissions) | | Hospitalized Non-Decedents (93,478 admissions) | |
| --- | --- | --- | --- |
| A419 (sepsis, unspecified)  R4182 (AMS, unspecified)  R0602 (shortness of breath)  J189 (pneumonia)  R531 (weakness)  N390 (UTI, site unspecified)  N179 (AKF, unspecified)  G20 (Parkinson’s disease)  J690 (aspiration pneumonitis)  R509 (fever, unspecified)  Total | 2,609 (8.3%)  2,278 (7.3%)  1,502 (4.8%)  1,272 (4.0%)  1,180 (3.8%)  1,059 (3.4%)  625 (2.0%)  617 (2.0%)  598 (1.9%)  570 (1.8%)  12,310 (39.2%) | R4182 (AMS, unspecified)  G20 (Parkinson’s disease)  R531 (weakness)  R0602 (shortness of breath)  A419 (sepsis, unspecified)  N390 (UTI, site unspecified)  J189 (pneumonia)  R55 (syncope and collapse)  R079 (chest pain)  R509 (fever, unspecified)  Total | 4,349 (4.7%)  3,941 (4.2%)  3,802 (4.1%)  3,324 (3.6%)  3,035 (3.2%)  2,877 (3.1%)  2,810 (3.0%)  2,503 (2.7%)  1,854 (2.0%)  1,524 (1.6%)  30,019 (32.1%) |

B. Principal Diagnoses

| Hospitalized Decedents (31,415 admissions) | | Hospitalized Non-Decedents (93,478 admissions) | |
| --- | --- | --- | --- |
| A419 (sepsis, unspecified)  J690 (aspiration pneumonitis)  N390 (UTI, site unspecified)  N179 (acute kidney failure)  G20 (Parkinson’s disease)  I130 (hypertensive heart/CKD)  J189 (pneumonia)  I110 (heart disease/failure)  T85311A (infection from indwelling urethral catheter )  I214 (NSTEMI)  Total | 4,886 (15.6%)  1,563 (5.0%)  1,162 (3.7%)  1,093 (3.5%)  1,076 (3.4%)  817 (2.6%)  796 (2.5%)  552 (1.8%)  443 (1.4%)  436 (1.4%)  12,824 (40.8%) | A419 (sepsis, unspecified)  G20 (Parkinson’s disease)  N390 (UTI, site unspecified)  N179 (acute kidney failure)  J189 (pneumonia)  J690 (aspiration pneumonitis)  J441 (COPD exacerbation)  J440 (COPD with LRI)  I130 (hypertensive heart/CKD)  I110 (heart disease/failure)  Total | 5,866 (6.3%)  5,415 (5.8%)  3,779 (4.0%)  2,265 (2.4%)  2,224 (2.4%)  1,594 (1.7%)  1,337 (1.4%)  1,241 (1.3%)  1,189 (1.3%)  1,167 (1.2%)  26,077 (27.9%) |

Abbreviations: AMS, altered mental status; UTI, urinary tract infection; AKF, acute kidney failure; CKD, chronic kidney disease; NSTEMI, non-ST-elevation myocardial infarction; COPD, chronic obstructive pulmonary disease; LRI, lower respiratory infection
